# Depolarization‐Based Multimodal Optical Imaging of Carious Lesions

**DOI:** 10.1002/jbio.202500422

**Published:** 2025-12-15

**Authors:** Julia Grundmann, Christian Hannig, Svea Steuer, Tobias Rosenauer, Lars Kirsten, Edmund Koch, Jonas Golde, Julia Walther

**Affiliations:** ^1^ TU Dresden, Faculty of Medicine Carl Gustav Carus Policlinic of Operative Dentistry, Periodontology and Pediatric Dentistry Dresden Germany; ^2^ TU Dresden, Faculty of Medicine Carl Gustav Carus Medical Physics and Biomedical Engineering Dresden Germany; ^3^ TU Dresden, Faculty of Medicine Carl Gustav Carus Anesthesiology and Intensive Care Medicine, Clinical Sensoring and Monitoring Dresden Germany; ^4^ Fraunhofer Institute for Material and Beam Technology IWS Dresden Germany

**Keywords:** caries, depolarization, microscopy, optical imaging, preventive dentistry, PS‐OCT

## Abstract

A comprehensive image catalog from an in vitro investigation of teeth with occlusal carious lesions is provided. The aim was to visualize carious lesions using various imaging techniques in order to present different stages of caries progression and to provide reference data for the development and validation of polarization‐sensitive optical coherence tomography (PS‐OCT) as a non‐ionizing diagnostic method for dental caries. The study covers a variety of approaches, bridging the gap from basic histopathological analysis of thin sections to future optical imaging of occlusal carious lesions in vivo. All measurements were performed to support the interpretation and validation of PS‐OCT imaging based on the degree of polarization (DOP).

## Graphical abstract

Occlusal caries is visualized using non‐ionizing imaging techniques, including polarization‐sensitive OCT (PS‐OCT). The image collection captures different stages of lesion development and supports the interpretation of optical signals by comparison with histology. The study lays the groundwork for future diagnostic tools that enable safe, accurate, and early caries detection without radiation.

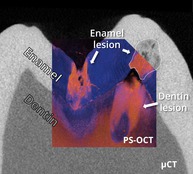

**Abbreviations**
DLM: depolarized light microscopy
DOP: degree of polarization
PLM: polarized light microscopy
PS‐OCT: polarization‐sensitive optical coherence tomography
μCT: X‐ray micro‐computed tomography

## 1 Introduction

### 1.1 Caries and Current Strategies in Caries Management

Untreated dental caries represents a significant global health issue. In 2017, 2.3 billion adults and 532 million children were affected, equating to a prevalence of 29.4% in permanent teeth and 7.8% in primary teeth [1]. As a result, dental caries remains the most common chronic disease in humans [2]. Caries is a disease that leads to the destruction of dental hard tissues, predominantly progressing from the occlusal and approximal tooth surfaces. It is biofilm‐dependent, multifactorial, and influenced by the patient's dietary behavior [3]. The pathomechanism of caries is still commonly described by the ecological plaque hypothesis introduced by Marsh in 1994 [4]. According to this concept, a microbial shift within the oral ecosystem leads to a predominance of pathogenic bacteria in the previously balanced biofilm [5]. A frequent intake of low molecular carbohydrates promotes the colonization and growth of cariogenic, acid‐tolerant bacteria within the oral biofilm [6]. During the metabolism of fermentable carbohydrates, biofilm‐residing bacteria generate organic acids, which lower the pH at the tooth surface and initiate enamel demineralization. This early, subclinical stage of mineral loss can be neutralized by the buffering capacity and ion reservoir of saliva [3]. However, when the buffering systems are overloaded, an imbalance in the de‐ and remineralization process develops [7]. As mineral loss continues, initial carious lesions become clinically visible as “white spots” on the enamel surface, although surface integrity remains intact at this stage [3]. Without preventive intervention, the progression of caries results in cavitation of the enamel surface. Once bacteria penetrate the dentin, the disease advances more rapidly than in enamel [8]. Research over the last century resulted in the rise of minimal intervention dentistry, an approach focusing on modifying the etiological factors of the caries process to preserve dental hard tissues [9]. The aim is to arrest existing lesions and prevent the formation of new lesions [3]. The “heal and seal” approach, known as modern caries management, includes non‐invasive therapy strategies such as biofilm control, the application of fluorides, and providing dietary guidance to the patient. Restoration of a caries lesion is only indicated after cavitation of the enamel surface and should be minimally invasive to further preserve dental hard tissue [8].

### 1.2 Diagnostic Techniques in Cariology

The detection of occlusal carious lesions and the decision regarding when and how to intervene in the dynamic caries process remain challenging for clinicians in most cases [8]. Visual inspection is the standard method for occlusal caries detection [10, 11]; however, its ability to identify non‐cavitated occlusal lesions is limited as demineralization can be clinically indistinguishable from extrinsic discoloration [12]. Furthermore, hidden caries, defined as a dentin lesion covered by clinically sound enamel, cannot be detected through visual inspection due to the absence of characteristic enamel changes [13, 14]. The International Caries Detection and Assessment System (ICDAS II) [15] has been shown to improve the accuracy and reproducibility of visual inspection by providing a standardized scoring system with a sensitivity of 85% and a specificity of 88% for dentin caries [16, 17]. The most important diagnostic tool to support visual inspection is digital bitewing radiography [18]. While often referred to as the diagnostic standard for approximal caries, bitewing radiographs have a relatively low specificity, potentially influencing treatment strategies [19]. Its diagnostic value for occlusal surfaces is further limited [10, 20]. Due to the superimposition of buccal enamel structures, early occlusal lesions often remain undetected until they have progressed into the dentin [21, 22]. Additionally, the depth of such lesions is frequently underestimated [23], and radiographs cannot reliably determine whether cavitation of the enamel surface has occurred [24]. Due to the exposure to ionizing radiation, the indication for radiographic imaging should be critically evaluated and aligned with the patient's individual caries risk profile [3]. Current diagnostic methods have methodological limitations, particularly in the detection and assessment of non‐cavitated occlusal lesions. Therefore, several non‐ionizing optical diagnostic techniques have been developed over the past decades [19]. Among these, near‐infrared transillumination (NIRT) with a wavelength of 780 nm, commercially available as DIAGNOcam (KaVo Dental, Biberach, Germany) [25], is currently the most commonly used in clinical practice and is supported by promising clinical data [12, 20, 26].

### 1.3 The Role of Polarization‐Sensitive Optical Coherence Tomography (PS‐OCT) in Dentistry

Another promising diagnostic technique is polarization‐sensitive optical coherence tomography (PS‐OCT); a high‐resolution, non‐ionizing, and non‐invasive imaging modality. Its underlying principle is analogous to ultrasound; however, instead of sound waves, it utilizes light to generate depth‐resolved cross‐sectional images of tissue structures [27, 28]. While its penetration depth is limited to approximately 2 mm, PS‐OCT offers a spatial resolution slightly lower but comparable to that of histological sections, depending on the microscope [29]. The technique enables real‐time acquisition of cross‐sectional images at a micrometer scale, providing information about tissue birefringence and reflectivity of the examined tissue [30, 31]. The diagnostic potential of PS‐OCT in dentistry, especially for the detection of initial carious lesions, has been extensively proven in the last 20 years [29, 32, 33, 34, 35, 36]. PS‐OCT offers high sensitivity and specificity for the detection of early carious lesions and enables three‐dimensional imaging of tooth structures [37]. However, its diagnostic accuracy can be limited by the scan‐direction‐dependent penetration depth and by multiple scattering beneath enamel lesions, which limits the evaluation of underlying dentin [38]. Despite its potential, to date, no commercial PS‐OCT system is available for clinical use [39]. Several companies, including Perceptive.io (USA) [40] and OssVis (South Korea) [41], are developing OCT‐based prototype systems for intraoral diagnostics. While promising in terms of technical feasibility and workflow integration, these systems have so far been described primarily in preclinical or industry‐based reports [42, 43]. However, different research groups provide promising data for approximal caries detection with OCT probes ex vivo [44, 45, 46] and in vivo [47] as well as for occlusal caries detection ex vivo [37].

### 1.4 Degree of Polarization (DOP) as a Diagnostic Parameter

Standard OCT systems typically provide intensity‐based cross‐sectional images and volumes. With PS‐OCT, at least the phase retardation is most often used as an indicator of tissue birefringence and changes in the microstructure [32]. The additional analysis of the degree of polarization (DOP) in the backscattered signal provides an intuitive contrast for detecting demineralized enamel [34, 36, 48] and other pathologically relevant changes, such as the formation of dental calculus [49]. Caries causes depolarization [33], that is, the randomization of the polarization state of incident light due to undirected backscattering. The resulting reduction in DOP offers complementary contrast and enables a more intuitive evaluation of PS‐OCT images than using only the intensity or retardation signal. It has been shown that DOP evaluation allows differentiation between surface staining and discoloration versus carious lesions [35]. Furthermore, DOP imaging has high sensitivity to demineralization and remineralization processes at the enamel surface [50]. In a previous publication related to this study, by the analysis of microscopic data combined with DOP evaluation, it was demonstrated that DOP imaging reliably indicates demineralization in enamel, while its diagnostic significance in dentin is less pronounced [51]. This demonstrates the need for basic research on the optical characteristics of enamel and dentin to allow valid interpretation of future in vivo PS‐OCT data.

The aim of this study was to visualize different stages of occlusal caries progression using multiple optical imaging modalities, including the evaluation of the degree of polarization (DOP). While the microscopic techniques applied have been described in detail elsewhere [51], the present work provides an overview of all imaging approaches, with a particular focus on PS‐OCT and its potential diagnostic relevance in clinical practice.

## 2 Materials and Methods

### 2.1 Experimental Design

**FIGURE 1:**
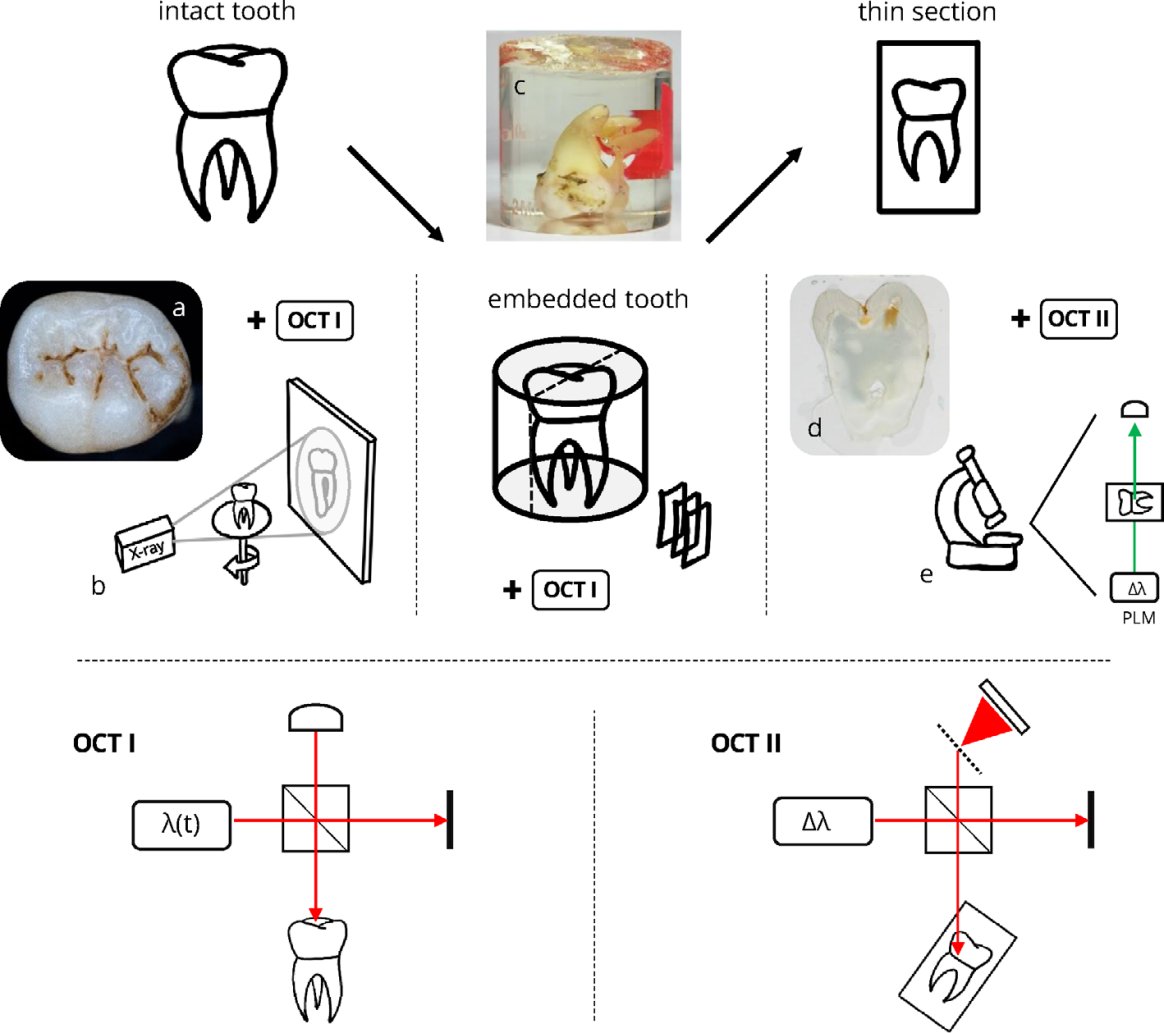
Schematic representation of the workflow used in this study with all applied optical imaging techniques. In the upper leftmost section of the figure, the procedures conducted on the intact teeth are depicted with (a) the occlusal photography of the intact tooth and (b) bitewing radiography. The central section of the figure illustrates the procedures conducted on the embedded teeth, shown in the (c) photography of the embedded tooth. The rightmost section of the figure displays the procedures conducted on the thin sections with a (d) photography of the thin section and (e) polarized light microscopy. Sketches of the wide‐field swept‐source PS‐OCT system (OCT I) used for imaging of the intact and embedded teeth as well as the high‐resoluton spectrometer‐based PS‐OCT system (OCT II) used for thin section imaging are depicted in the bottom row. Adapted with permission from Grundmann et al. [51] Optica Publishing Group.

As shown in Figure 1, the study was carried out in three successive steps to ensure the precise alignment of volumetric and thin sectional data within the same sectional plane. Accordingly, data acquisition was performed under three different conditions of the carious teeth: intact teeth, embedded teeth, and thin sections. All methods were carried out in accordance with relevant guidelines and regulations.

### 2.2 Examinations of the Intact Teeth

A total of 17 teeth were included in the study. In accordance with the guidelines of the Central Ethics Committee of the German Medical Association, the use of teeth extracted for medical reasons does not require separate ethical approval for research purposes [52]. A photograph of the occlusal surface and a digital bitewing radiograph were taken of each tooth, representing the conventional diagnostic techniques (visual inspection and digital bitewing radiography). For validation, X‐ray micro‐computed tomography (μCT) images were acquired and served as the 3D radiographic reference procedure. The μCT volumes were acquired on the intact, non‐embedded teeth using a μCT specimen scanner (SCANCO vivaCT 40, SCANCO Medical AG, Brüttisellen, Switzerland) at the Bone Lab Dresden. All μCT scans were recorded with the following parameters: voltage: 70 kV, exposure time: 0.35 s, current: 0.114 mAs. The voxel size is 10.5 μm × 10.5 μm × 10.5 μm. Subsequently, the intact teeth were examined with PS‐OCT from occlusal scan direction. All volumes were acquired using an in‐house developed wide‐field swept‐source PS‐OCT system (OCT I in this study) with a tunable laser (AXP50125‐6, Axsun Technologies Inc., Billerica, Massachusetts) as a swept light source, the setup of which is described elsewhere [48]. The only change made in comparison to the previously published setup was a frequency‐doubling of the k‐clock signal, resulting in a doubling of the available imaging depth. This enabled imaging the entire occlusal surface of the samples within the available depth range, as pits and fissures often show topographic differences of more than 5 mm. However, the penetration depth in enamel remained in the range of approximately 2 mm assuming a refractive index at 1300 nm of 1.6 for enamel. The following measurement parameters were used: A‐scan rate: 50 kHz, central wave length: 1310 nm, band width: 110 nm, optical power on sample: 8.5 mW, lateral step size: 8 μm, volume size: 1408 × 1408 A‐scans, imaging depth: 9.8 mm, axial resolution in air: 15 μm and lateral resolution: 16 μm.

### 2.3 Embedding Process

After completion of the examination of the intact teeth, all teeth were embedded in light‐curing resin standing on the occlusal surface. Before embedding, the teeth were fixed, drained and infiltrated. The teeth were fixed with formalin 4% for 24 h and then rinsed with distilled water for 10 min. This was followed by dehydration in an ascending ethanol series for 12 h. Using a combination of absolute ethanol/Technovitő 7200 VLC 1:1, preinfiltration was carried out for 60 min. Subsequently, the teeth were infiltrated with Technovitő 7200 VLC for 60 min. Each tooth was fixed standing on the occlusal surface at the bottom of a sample container with light‐curing precision adhesive. After polymerization of the precision adhesive for 10 min, the sample vessel was filled with Technovitő 7200 VLC. Subsequently, the light‐curing resin was polymerized for 4 h. It was then possible to perform a PS‐OCT scan (OCT I) of the resin blocks with embedded teeth as a reference for subsequent reconstruction of the thin section position. It is important to note that the PS‐OCT volume of the embedded tooth therefore served as a reference image throughout the entire experimental setup, but is not shown here. After reviewing the μCT images and the PS‐OCT reference image, a grinding direction was determined and noted for each tooth. The thin sections were then prepared with the cutting direction selected at exactly 90° to the embedded occlusal surface using the thin‐section technique by Donath with a thickness of approximately 80 μm [53].

### 2.4 Examination of the Thin Sections

For each tooth, one to two representative thin sections were selected which best depicted the carious lesion center. In this study, 24 thin sections were thus obtained. The selected thin sections were examined with a commercially available spectrometer‐based PS‐OCT system (TEL220PSC2, Thorlabs GmbH, Dachau, Germany; OCT II in this study) equipped with a high‐resolution objective (LSM02, Thorlabs). In comparison to the system used for imaging the intact teeth, where a large imaging depth was required, here an almost microscopic resolution was achieved for the analysis of the thin sections. The following parameters were used: A‐scan rate: 48 kHz, central wave length: 1300 nm, band width: 247 nm, optical power on sample: 2 mW, lateral step size: 3 μm, imaging depth: 3.5 mm, axial resolution: 5.5 μm and lateral resolution: 7 μm. As a histological gold standard, the thin sections were examined with a polarized light microscope with three images being taken of each section: conventional polarized light microscopy, polarized light microscopy with an additional full‐wave plate and conventional transmitted light microscopy. As a final investigation, the DOP was evaluated by a newly developed technique based on a modification of the polarized light microscope, resulting in the introduction of depolarized light microscopy. Further details on the microscopic setup and depolarized light microscopy (DLM) can be found in a separate publication related to this study [51].

### 2.5 Data Alignment

As described previously [54], custom‐developed MATLAB (Version R2022b, MathWorks Inc., Natick MA, USA) scripts were used for data processing and DOP calculation. Semi‐automatic registration of the volumetric and thin sectional data and subsequent analysis of the datasets in the same sectional plane were likewise carried out using custom‐developed MATLAB scripts. As described by Golde et al. [36], the semi‐automatic registration of PS‐OCT and μCT volumes was realized using a manual pre‐alignment and an automatic optimization based on 3D image processing. For pre‐alignment, a MATLAB implementation of Horn's method that allows pairwise selection of corresponding coordinates within both volumes followed by three‐dimensional alignment [36] was used for coarsely adjusting the spatial position of both volumes. Optimizing this initial result, a 3D Canny edge detection as well as the iterative closest point (ICP) algorithm, based on the MATLAB Computer Vision Toolbox, were used for fine‐tuning the registration.

The first step entailed the registration of the embedded PS‐OCT image, which served as the reference image, with the μCT dataset for each tooth. The registration was also performed for the PS‐OCT image from the occlusal perspective on the non‐embedded tooth with the μCT volume. After preselecting coordinates within the PS‐OCT and μCT volume data in prominent regions on the occlusal surface, that is, cusp tips and fissures, the 3D alignment was achieved using the aforementioned ICP algorithm supplemented by Horn's method. This dual registration onto the μCT data enabled the visualization of the non‐embedded PS‐OCT data in the exact same cross‐sectional plane as the histological sections in the subsequent steps, as the key challenge of visualizing all optical imaging techniques in a single plane is aligning the volumetric data with the cross‐sectional image data in the same plane.

The next step involved locating the cross‐sectional plane of the thin section within the volumetric PS‐OCT and μCT data. To identify the section plane, the registered PS‐OCT of the embedded tooth and the corresponding μCT data were viewed in direct comparison with the microscopy image of the thin section. Owing to the consistent orthogonal orientation in which all thin sections were prepared, it can be ascertained that the section plane can be reliably identified by a 2D rotation in the enface plane of the embedded PS‐OCT volume and a subsequent fly‐through of the axial‐lateral planes in comparison to the microscopy images. To further validate this process, the transmitted light images were subsequently imported, and the matching with the μCT planes was confirmed by aligning preselected coordinates within the thin section, that is, cusp tips and the DEJ. Finally, the registration of the volume data and thin section images was accomplished and all images were exported for visualization and evaluation within the same sectional plane.

## 3 Results

**FIGURE 2:**
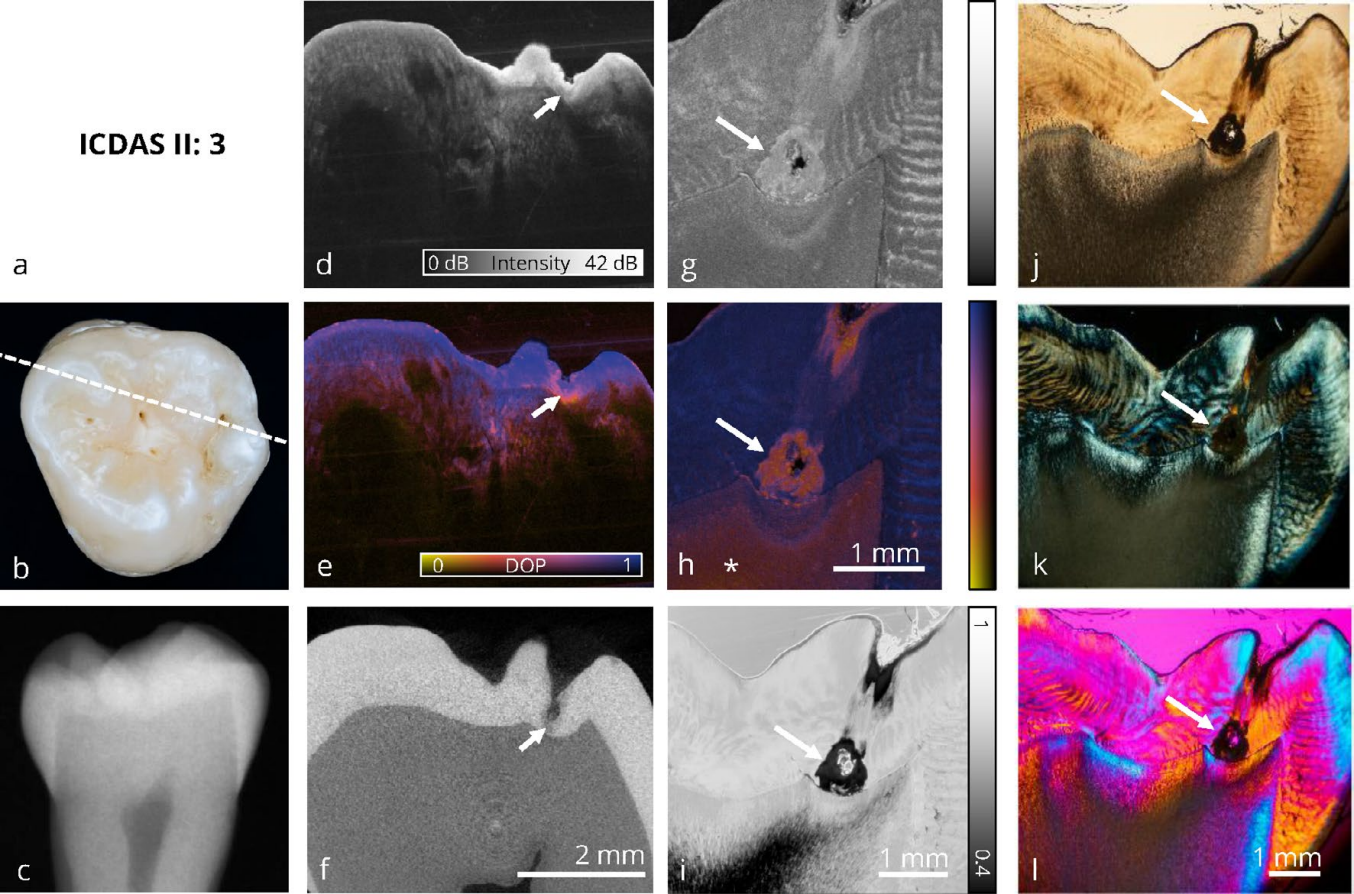
Collection of optical imaging data of an occlusal carious lesion in the same sectional plane. In this image, a clinically healthy tooth shows signs of early demineralization (→). At this stage, no therapeutic intervention is required, as the lesion can be monitored over an extended period. The benefit of using PS‐OCT with degree of polarization (DOP) representation (e), compared to intensity based imaging (d), is evident in the detection of these initial changes, as the DOP contrast provides better delineation of the initial lesion without highlighting the entire surface. Additionally, the PS‐OCT thin‐section with DOP‐imaging (h) reveals that healthy dentin has depolarizing properties (*). As suspected, digital bitewing x‐ray (c) shows no pathological findings. (a) ICDAS II classification, (b) photograph of the occlusal surface with marked slicing region, (c) bitewing x‐ray, (d) PS‐OCT intensity volume (OCT I), (e) PS‐OCT DOP volume (OCT I), (f) μCT volume, (g) PS‐OCT intensity thin section (OCT II), (h) PS‐OCT DOP thin section (OCT II), (i) depolarized light microscopy, (j) transmitted light microscopy, (k) polarized light microscopy, (l) polarized light microscopy with additional full‐wave plate. Panels (d)–(f), (g) + (h), and (j)–(l) share the same scale bar.

**FIGURE 3:**
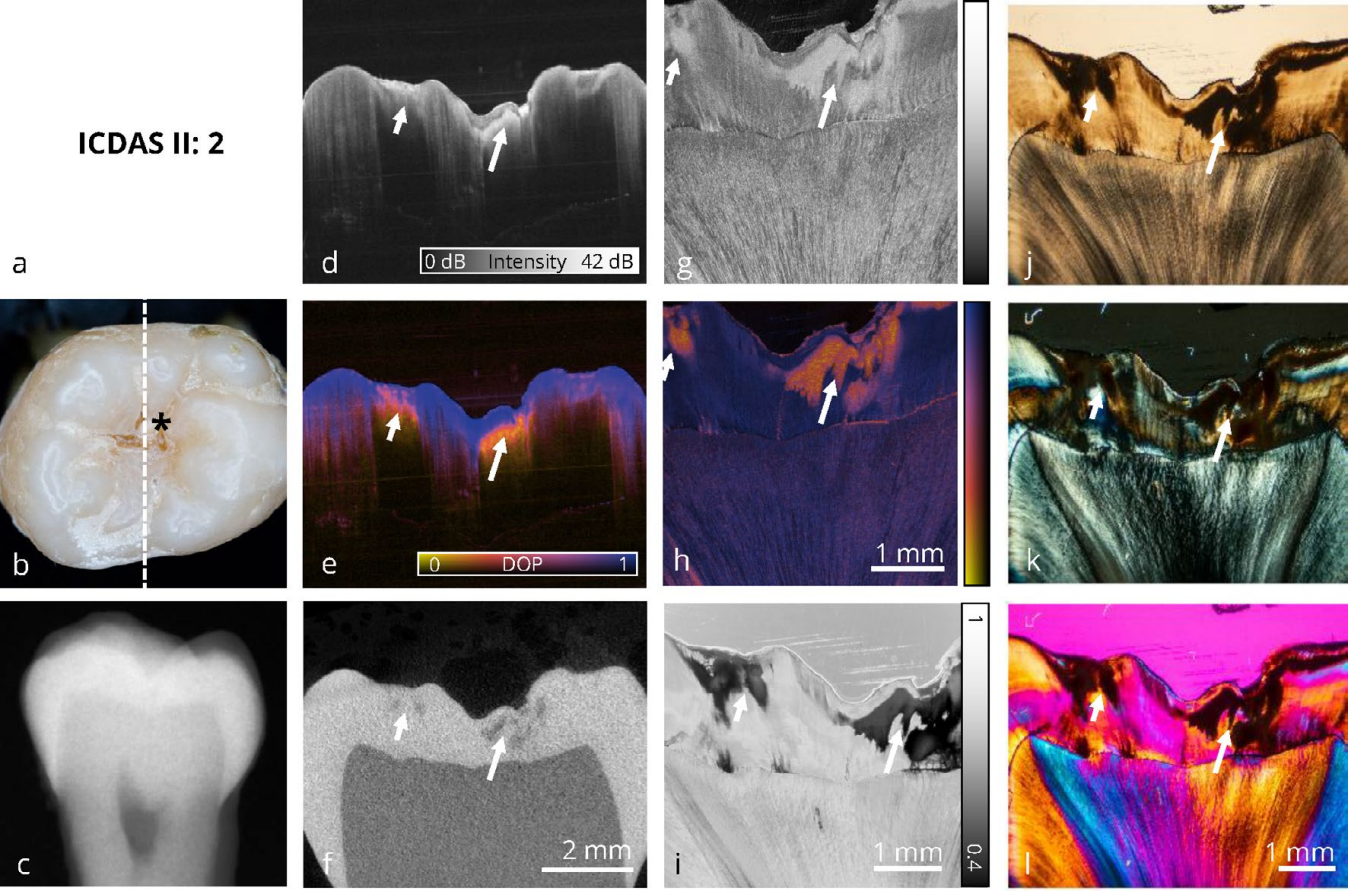
Collection of optical imaging data of an occlusal carious lesion in the same sectional plane. Calculus (*) is visible on the occlusal surface. Differentially, the structure on the occlusal surface may also represent a discoloration or an incipient carious lesion, while typical clinical signs of occlusal caries are absent. Thin sections reveal the presence of an initial enamel demineralization (→) that requires further monitoring. This early‐stage lesion is also clearly detected in the PS‐OCT volume, visible in both intensity (d) and DOP (e) imaging, where the DOP representation allows a more distinct visualization. PS‐OCT intensity imaging additionally reveals the presence of a surface deposit. In contrast, the digital bitewing x‐ray shows no abnormalities, showing the limitations of conventional radiographic diagnostics in the detection of early stage occlusal caries lesions. Refer to Figure 2 for a detailed enumeration.

**FIGURE 4:**
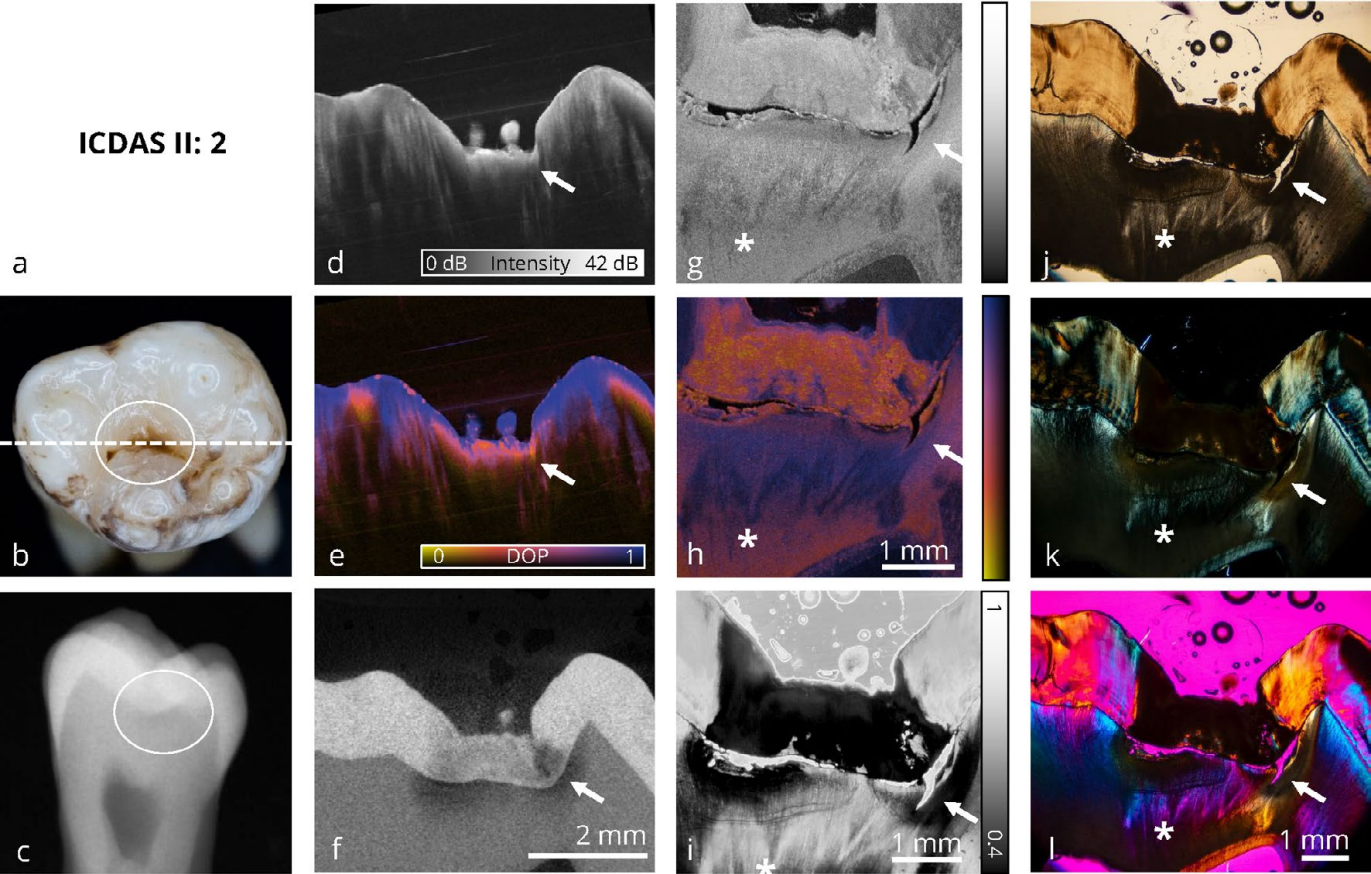
Collection of optical imaging data of an occlusal carious lesion in the same sectional plane. The occlusal surface shows brownish discoloration and visible signs of demineralization (white circle). The enamel surface remains intact and shows no signs of cavitation. In the conventional radiograph (c), an occlusal carious lesion extending into the dentin is visible (white circle). Corresponding thin‐section images confirm the progression of the demineralization into the dentin (*). Depending on the patient's clinical symptoms, continued monitoring of the lesion (→), combined with preventive measures, is recommended. The direct comparison of PS‐OCT intensity (d) and PS‐OCT DOP (e) volume data demonstrates the value of DOP‐based visualization, as it provides an intuitive and clearer representation lesion's extent. PS‐OCT may underestimate the full extent of the lesion's progression into dentin due to multiple scattering within the enamel. Refer to Figure 2 for a detailed enumeration.

**FIGURE 5:**
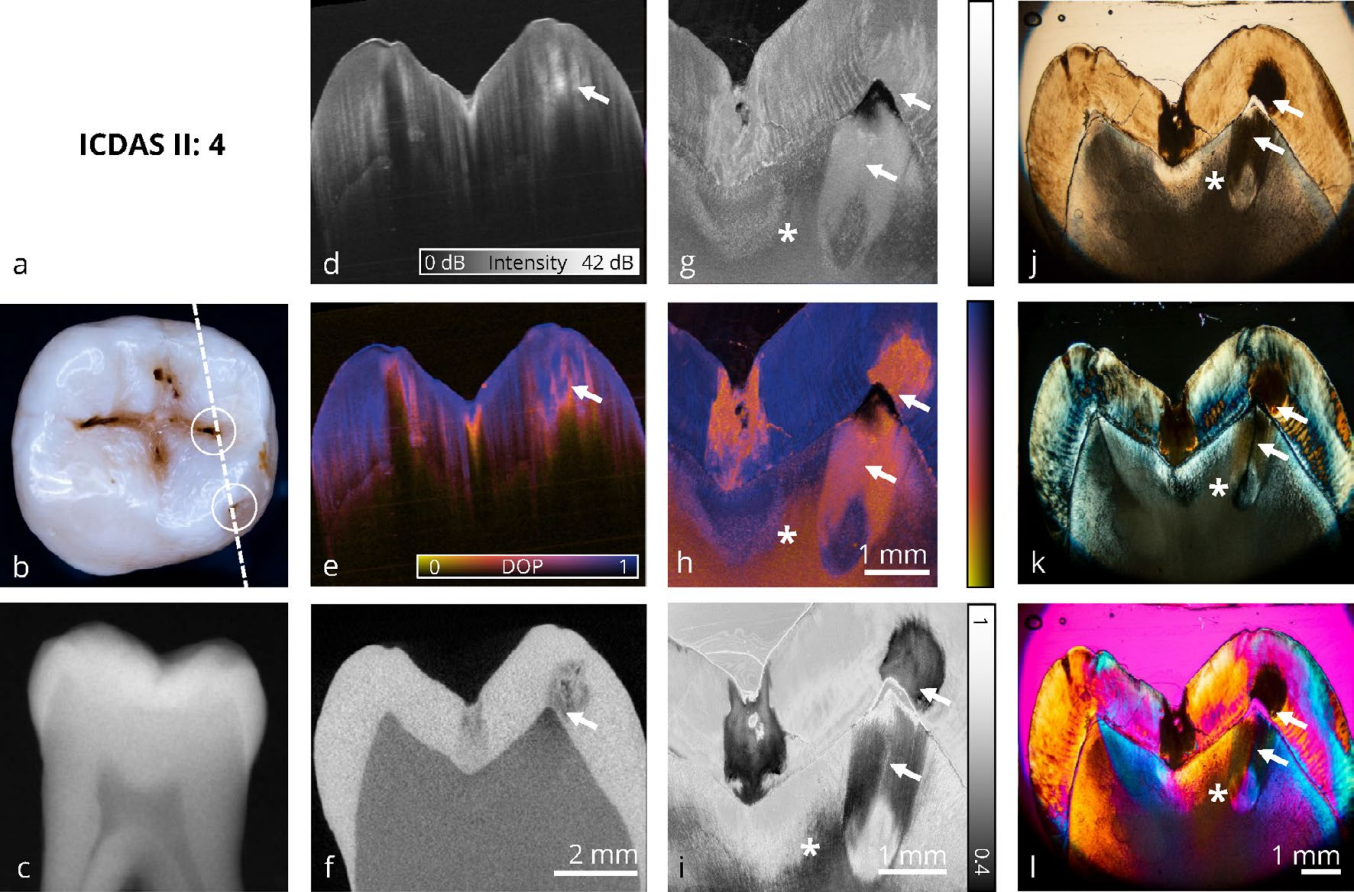
Collection of optical imaging data of an occlusal carious lesion in the same sectional plane. The fissures show brownish discoloration with two minor cavitations visible in the cross‐section area (white circles). Clinically, it is difficult to determine whether the lesion (→) is limited to the enamel or has progressed into dentin. Additionally, the digital bitewing radiograph does not allow for a reliable diagnosis. PS‐OCT clearly reveals the demineralizations in enamel (d, e), with μCT data confirming the extent of the lesion, but has only limited interpretability in dentin when the light is incident from the occlusal direction. Due to initial cavitation, minimally invasive treatment is indicated. Thin sections (h, i) show low DOP values in both demineralized (→) and healthy dentin (*), which indicates the depolarizing properties of these areas. The comparison of PS‐OCT cross‐sectional (h) and volumetric (e) data shows that carious lesions can be reliably identified using this technique. However, interpretation may be affected by the angle of incident light and the limited penetration depth of the PS‐OCT setup. Refer to Figure 2 for a detailed enumeration.

**FIGURE 6:**
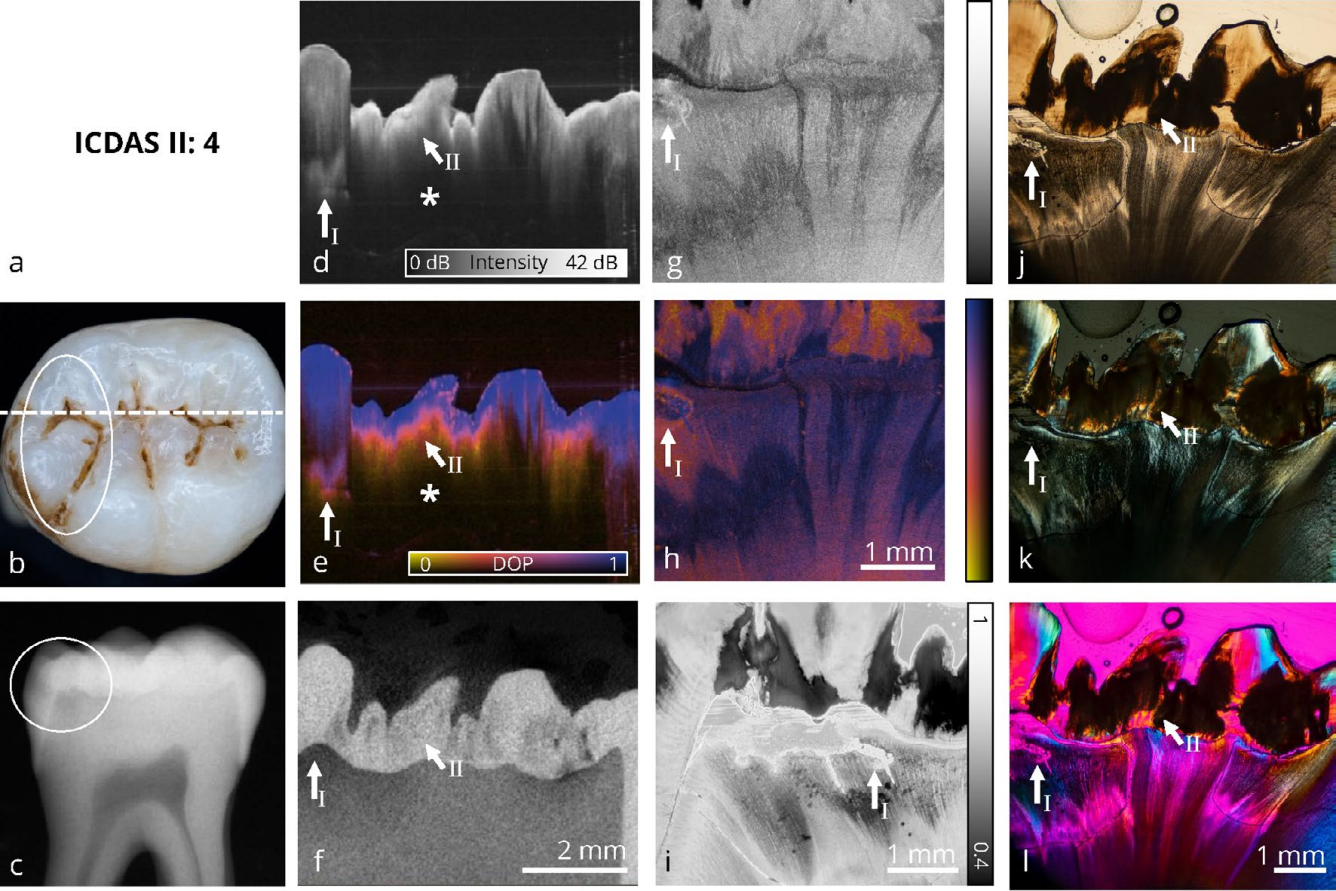
Collection of optical imaging data of an occlusal carious lesion in the same sectional plane. The occlusal surface shows characteristics of an active caries lesion, including discolored fissures with opaque cuspal inclines. However, the lesion depth is difficult to assess clinically. The grayish translucency on the left side of the occlusal photograph (b, white circle) is a sign for deeper dentin involvement, as confirmed by the digital radiograph (c, white circle) and the thin sections. Although no macroscopic cavitation is present, a minimally invasive restoration is indicated, as a small, clinically undetectable cavitation in the occlusal surface is likely. The PS‐OCT volumetric data (d, e) demonstrates limited sensitivity for the extension into dentin with the case of a more severe lesion in the dentin (→ I) as well as a lesion, which is primarily indicated in the enamel (→ II). Furthermore, multiple scattering in the enamel reduces light penetration into the dentin (*) and results in a tendency to overestimate the lesion depth. Refer to Figure 2 for a detailed enumeration.

**FIGURE 7:**
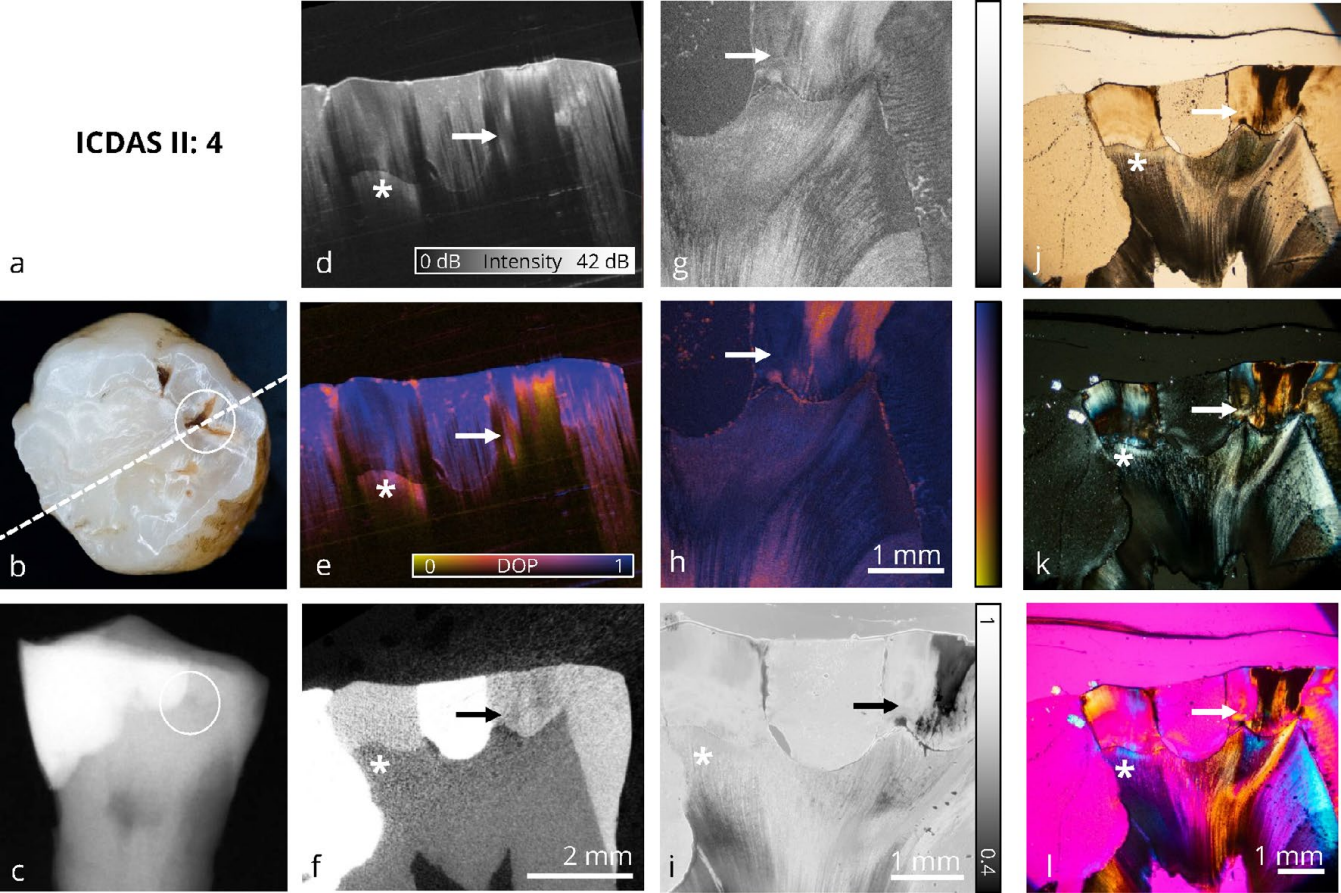
Collection of optical imaging data of an occlusal carious lesion in the same sectional plane. This image panel displays a tooth with an extensive composite restoration. Adjacent to the restoration margin, a discolored fissure is visible (b, white circle), which corresponds to a demineralized area (→) extending into the outer third of dentin in the PS‐OCT volume (d, e). In contrast, the radiograph does not reveal a clear lesion (c, white circle). Clinically, a localized repair restoration would be indicated, as demineralized enamel does not represent an appropriate restoration margin. The composite material exhibits a DOP value of 1 (e, i). The region at the dentin‐enamel junction (DEJ) adjacent to the composite restoration (*) shows a reduced DOP signal in both DLM thin section (i) and PS‐OCT DOP volume imaging (e), whereas the μCT scan shows no signs of structural alteration in this area. Refer to Figure 2 for a detailed enumeration.

The image panels are structured into initial, enamel‐limited (Figures 2 and 3) and advanced (Figures 4, 5, 6, 7) carious lesions affecting dentin. Therefore, a representative selection of six teeth with occlusal carious lesions at different stages of progression is presented from a total of 17 examined molar teeth. For each tooth, all optical imaging data are displayed within an image panel, allowing for direct comparison of the various co‐registered optical imaging techniques within the exact same cross‐sectional plane, structured as follows: the far‐left column shows conventional diagnostic methods; visual inspection using ICDAS classification and digital bitewing radiography. The second column from the left presents cross sections of volumetric data obtained by wide‐field PS‐OCT and micro‐computed tomography (μCT). The second column from the right displays the corresponding thin section analyzed by high‐resolution PS‐OCT and depolarized light microscopy (DLM) [51]. The far‐right column contains conventional microscopic techniques: transmitted light microscopy, polarized light microscopy (PLM) and polarized light microscopy with an additional full‐wave plate. A clinical interpretation is provided for each tooth from both a diagnostic and therapeutic perspective.

### 3.1 Visualization of Initial Carious Lesions

Figures 2 and 3 represent initial carious lesions with no need for therapeutic intervention.

In Figure 2, a tooth with early signs of demineralization is shown. While the visual inspection results in an ICDAS code 3, suggesting a potential overestimation of the lesion severity, the corresponding conventional radiograph (c) reveals no signs of demineralization. In contrast, the wide field PS‐OCT volumetric dataset, validated by corresponding μCT data (f), demonstrates a localized reduction in the degree of polarization (DOP) indicative of early demineralization. DOP‐based PS‐OCT (e) provides a more intuitive interpretation of demineralized zones compared to conventional intensity‐based PS‐OCT (d) imaging. Here, the perspective diagnostic benefit of PS‐OCT in comparison to conventional methods are demonstrated: the lesion is detected without being overestimated as with visual inspection, and PS‐OCT closes the diagnostic gap left by radiographic imaging. Interpretation of the corresponding high‐resolution thin‐section PS‐OCT data (g, h) further demonstrates the diagnostic potential of DOP contrast compared to conventional intensity‐based imaging, as the initial lesion is entirely visualized using DOP‐based imaging techniques on thin sections (PS‐OCT (h) and DLM (i)). A comparison between DLM and conventional polarized light microscopy (PLM, k) further confirms the diagnostic advantage of DOP‐based imaging, as the demineralization in enamel can be clearly delineated in DLM (i) compared to PLM (k). Healthy dentin exhibits a reduced DOP in the PS‐OCT thin section and DLM (h, i). In this case, PS‐OCT enables the earliest detection of a carious lesion and effectively bridges the diagnostic limitations of visual and radiographic methods. From a clinical point of view, no therapeutic intervention is indicated; continued monitoring is recommended.

Similar to the first tooth, Figure 3 visualizes a nearly sound tooth exhibiting discoloration within the fissures on the occlusal surface, classified as ICDAS code II. Based on visual inspection (b), the fissure alteration could represent staining, calculus, or an initial stage of demineralization, although typical carious alterations such as opacities near the fissures are not present. The bitewing radiograph (c) is clinically unremarkable. Volumetric wide‐field PS‐OCT data (d, e) indicate initial enamel demineralization, which appears more obvious in comparison to the reference μCT dataset (f). Notably, the surface deposit is visible in the intensity‐based imaging (d), as well as in the DOP image (e) as a blue surface layer above the demineralization. Again, DOP imaging yields a more intuitive contrast relative to intensity‐based imaging. The extent of the lesion becomes clearly visible in the thin sections analyzed by high‐resolution PS‐OCT (g, h) and depolarized light microscopy (DLM, i). Here, in contrast to Figure 2, healthy dentin does not show a depolarization signal. Once again, DLM demonstrates advantages over conventional PLM (k) in the interpretation of thin sections, as the grayscale visualization of the degree of polarization (DOP) enables the immediate identification of demineralized areas in enamel. As with Figure 2, no therapeutic intervention except removal of the calculus is indicated.

In the presented image panels, PS‐OCT, particularly in combination with DOP evaluation, allows the detection of these early enamel demineralizations. Lesions at an initial, subclinical stage are visualized in both PS‐OCT intensity volumes and corresponding DOP representations, whereas the DOP imaging approach allows a more intuitive interpretation. Therefore, PS‐OCT offers superior sensitivity in the visualization of non‐cavitated incipient lesions compared to conventional bitewing radiographs in the here presented image panels and can bridge the radiographically “blind” area.

### 3.2 Visualization of Carious Lesions at an Advanced Stage

Figures 4, 5, 6, 7 visualize carious lesions at an advanced stage, characterized by dentin involvement prior to cavitation of the enamel surface. These cases are located at the diagnostic threshold for operative intervention, as conventional methods such as visual inspection and bitewing radiography do not reliably reflect the true extent of the lesion. This can result in premature or delayed therapeutic decisions.

In Figures 4, 5, and 7, the extent of the lesion is underestimated in bitewing radiographs when compared to the ex vivo reference method, μCT, and furthermore PS‐OCT as well as the four microscopic techniques applied to thin sections. In these cases, visual inspection based on ICDAS criteria combined with bitewing radiography does not provide sufficient information to support therapy planning, whereas PS‐OCT reliably identifies the presence of the lesion. Depending on the patient's symptoms, preventive measures are indicated in Figure 4, whereas in Figures 5 and 7, a restorative treatment is necessary. In contrast, Figure 6 presents a tooth with characteristics of an active carious lesion, including a grayish shadow visible beneath the enamel (Figure 6b) and a distinct radiolucency on the bitewing radiograph at the corresponding site (Figure 6c). Here, a restorative approach is indicated due to the presence of a potential cavitated entry point.

Benefits and limitations of the volumetric wide‐field PS‐OCT (OCT I) data for diagnosing advanced caries lesions are demonstrated. When examining the PS‐OCT volume data, it becomes evident that depolarization imaging detects demineralization better than intensity imaging. The DOP contrast enables the carious lesions to be identified at first sight and provides an increased value compared to intensity imaging in PS‐OCT, as it can be seen in Figures 4 and 6. Furthermore, surface‐proximate areas tend to be overestimated within the intensity‐based representation, whereas DOP imaging allows for a more precise localization of demineralized areas (Figures 4 and 6). In Figure 4, and similarly in Figure 6, PS‐OCT (d, e) appears to underestimate lesion depth. A likely explanation is the extensive demineralization within the enamel, which causes multiple scattering and hinders optical penetration into dentin, particularly near the dentin‐enamel junction. Additionally, Figure 6 illustrates that the scan orientation can influence lesion visualization with PS‐OCT. However, the observed slight misalignment in the left part of the image may also result from a local mismatch in the refractive index, causing an apparent shift of the dentin‐enamel junction (DEJ) to greater depth due to a longer optical path. This should be taken into account when interpreting lesion depth in PS‐OCT images.

Furthermore, PS‐OCT based depolarization imaging is comparable to depolarized light microscopy. In direct comparison with DLM (i), it is shown that the contrast based on depolarization is reliable for detecting carious lesions in enamel (Figures 4, 5, 6). In DLM thin sections, reduced DOP values lower than 0.5 are represented as black, whereas in high‐resolution PS‐OCT (OCT II) thin sections, they are shown as orange. From a histological perspective, depolarization imaging based on PS‐OCT is directly comparable to microscopic depolarization imaging (DLM). High‐resolution PS‐OCT (OCT II) also identifies demineralized areas within dentin when data is acquired on thin sections, as it can be seen in Figures 4, 5, 6. This suggests that the limited representation of carious lesions within volumetric wide‐field PS‐OCT (OCT I) data is primarily attributed to scan direction and limited penetration depth. Based on the presented image panels, PS‐OCT is suitable for the detection and monitoring of carious lesions at an advanced stage. However, scan orientation affects the optical penetration depth and may influence lesion visualization.

## 4 Discussion

The aim of this study was to investigate the effectiveness of DOP‐based PS‐OCT imaging for detecting both initial and advanced carious lesions. A particular strength of the dataset is the co‐registered visualization of multiple optical imaging modalities within the exact same cross‐sectional plane. This alignment was achieved through precise spatial registration and allows a direct comparison of the optical properties of dental hard tissues across modalities, which contrasts with previous work [37, 44, 45, 46, 47] where imaging was often performed in non‐identical or adjacent planes.

The feasibility of polarization‐sensitive optical coherence tomography (PS‐OCT) to detect early‐stage lesions is well established [29, 31, 32, 39]. As previously shown, evaluating carious lesions based on the degree of polarization (DOP) is a promising diagnostic approach [33]. In particular, PS‐OCT with DOP imaging allows intuitive identification of carious lesions compared to intensity‐based imaging [34, 35, 36]. In the present study, histological thin sections were examined using high‐resolution PS‐OCT (OCT II) and compared with volumetric wide‐field PS‐OCT (OCT I) data. The findings demonstrate that PS‐OCT can reliably detect carious changes in dental hard tissues, although image interpretation may depend on the angle of incident light. Initial carious lesions were consistently detected (see Figures 2 and 3) and could be distinguished from discolorations and calculus. Regarding the detection of advanced occlusal carious lesions, our findings expand upon previous work by Shimada et al. [37], particularly in terms of lesion detection and interpretation of PS‐OCT data for occlusal caries under ex vivo conditions. As shown in the microscopic evaluation [51], the interpretation of PS‐OCT data in both thin sections and volumetric datasets confirms that, in enamel, depolarization is indicative of demineralization. In dentin, however, this relationship is not consistently observed, as healthy dentin also exhibits depolarizing properties (see Figures 2 and 5). The image panels demonstrate that wide‐field PS‐OCT with DOP imaging is particularly useful in cases where lesions are not yet detectable on radiographs but require monitoring to avoid unnecessary invasive treatment decisions. Furthermore, as outlined in Figures 4, 5, and 7, the added value of PS‐OCT lies in its potential to complement conventional diagnostic procedures, particularly by helping to clarify borderline findings in situations of clinical uncertainty [39]. Here, the DOP contrast allows for a more intuitive interpretation of the PS‐OCT data compared to intensity‐based imaging. However, it is important to note that in cases of deeper lesions with demineralization below the dentin‐enamel junction (DEJ), as shown in Figure 6, interpretation may be limited. This is due to the angle of incident light [38] or multiple scattering caused by demineralization in enamel. Although there are no recently published clinical studies investigating the feasibility of PS‐OCT for detecting occlusal carious lesions in vivo, the high sensitivity for detecting initial carious lesions [39], as well as the ability to visualize lesions affecting the DEJ as demonstrated in this study, make PS‐OCT a promising tool for future non‐ionizing caries diagnostics. This potential is already reflected in the ongoing development of several OCT systems designed for preclinical in vivo use [41, 42].

The limitations of this study concern the small sample size and the qualitative design of the study. Due to extensive data processing, especially data registration and precise localization of the plane section, the study was limited to a small sample size to present a high‐quality image catalog. Consequently, no statistical statements regarding the utility of PS‐OCT for the detection of occlusal caries can be made. Therefore, the conclusions presented in the results section are limited to a qualitative interpretation of the image panels and cannot be generalized. As the study was carried out in vitro, the actual clinical conditions are not accurately represented during data acquisition. This relates to the fact that in vivo, teeth are: (a) surrounded by saliva, (b) coated with a pellicle on the surface, and (c) often covered by a mature biofilm, which has developed from the pellicle [55]. Biofilm predominantly accumulates at predilection sites, which are especially the fissures on the occlusal surface and the interproximal surfaces. Due to the fact that this study was conducted on cleaned teeth in vitro, the influence of these organic components was not taken into account. Furthermore, the environment in an oversaturated solution of calcium and phosphate, such as saliva, differs significantly from the conditions in the laboratory where teeth are thoroughly cleaned and stored in thymol solution. Additionally, an overestimation of the depolarization signal may be present in the PS‐OCT thin sections due to the dry state of the teeth after the embedding process [35].

In summary, this study paves the way from basic research involving microscopic evaluation of thin sections to future in vivo imaging of occlusal caries lesions. By integrating conventional and advanced polarized light microscopy, digital bitewing radiography, μCT and PS‐OCT, the presented data set bridges the gap towards future caries diagnosis with PS‐OCT in clinical dentistry. All measurements were performed to support the interpretation and validation of PS‐OCT based caries detection with DOP imaging. An image catalog visualizing different stages of the carious process is provided, serving as a reference for both research and dental education. Furthermore, clinicians can utilize this image catalog for educational purposes, as it provides a diverse set of cases for training and diagnosis.

## 5 Conclusion

The co‐registered image panels presented in this study demonstrate that PS‐OCT combined with DOP evaluation represents a valuable and promising approach not only for the detection of initial carious lesions, but also for the monitoring of advanced lesion progression. Nevertheless, further in vivo studies are required to advance PS‐OCT as a diagnostic tool for the detection and monitoring of occlusal caries lesions and to identify potential limitations under clinical conditions.

## Author Contributions

**Julia Grundmann:** data acquisition, data analysis, validation, writing, figure creation. **Christian Hannig:** project conception, data analysis, editing. **Svea Steuer:** data acquisition, data analysis, writing, figure creation. **Tobias Rosenauer:** data acquisition. **Lars Kirsten:** resources, editing. **Edmund Koch:** project conception. **Jonas Golde:** project conception, data acquisition, data processing, validation, writing, figure creation. **Julia Walther:** project conception, editing. All authors reviewed and approved the final manuscript. Jonas Golde and Julia Walther share senior authorship.

## Funding

The authors have nothing to report.

## Conflicts of Interest

The authors declare no conflicts of interest.

## Data Availability

Available datasets generated and analysed during the current study include μCT scans [56], raw and processed images from polarized [57] and depolarized [58] light microscopy, as well as occlusal surface photographs and bitewing radiographs of all 17 teeth [59] in the OpARA (Open Access Repository and Archive, hosted by TU Dresden, Dresden, Germany) repository at https://opara.zih.tu‐dresden.de. The PS‐OCT data, including volumetric and thin‐sectional datasets, generated and analyzed during the current study is not publicly available at this time due to institutional restrictions regarding the publication of data processing methods, but is available from the Department of Medical Physics and Biomedical Engineering (julia.walther@tu-dresden.de) on reasonable request.
